# Association between triglyceride glucose-body mass index and outcomes in patients with acute ischemic stroke: a retrospective secondary analysis of a prospective Korean cohort

**DOI:** 10.3389/fneur.2026.1806171

**Published:** 2026-05-29

**Authors:** Mingxing Lou, Changchun Cao, Jie Jia

**Affiliations:** 1 Department of Rehabilitation Medicine, The First Affiliated Hospital of Fujian Medical University, Fuzhou, Fujian, China; 2 Fujian Branch of Huashan Hospital, Fudan University, Fuzhou, Fujian, China; 3 National Clinical Research Center for Aging and Medicine, Huashan Hospital, Fudan University, Shanghai, China

**Keywords:** acute ischemic stroke, nonlinear relationship, restricted cubic spline, triglyceride glucose-body mass index, unfavorable outcomes

## Abstract

**Background:**

The triglyceride glucose-body mass index (TyG-BMI) has recently garnered attention as a robust surrogate indicator for insulin resistance (IR). However, despite this burgeoning interest, the prospective effect of TyG-BMI on adverse clinical outcomes in acute ischemic stroke (AIS) individuals remains incompletely understood. Consequently, this investigation seeks to elucidate the effect of TyG-BMI on 90-day poor outcomes in AIS individuals.

**Methods:**

During the period of January 2010 to December 2016, this longitudinal investigation encompassed 1,722 individuals diagnosed with AIS who were treated at Seoul National University Hospital (monocentric) in Korea. Blood samples were collected at admission, and all patients were admitted within 7 days after stroke onset. Binary logistic regression analysis was subsequently utilized to evaluate the effect of TyG-BMI on adverse outcomes observed at 90 days. Potential nonlinear dose–response associations were employed using restricted cubic spline (RCS) models to identify threshold (inflection) effects.

**Results:**

The final analysis included a total of 1,722 individuals, with males constituting 61.56% of the cohort. Among these participants, 393 (22.82%) were below the age of 60, 457 (26.54%) were aged between 60 and 70 years, 610 (35.42%) fell within the 70 to 80-year age bracket, and 262 (15. 21%) were over 80 years of age. The median admission NIHSS score was 3 (1-7). In multivariable logistic regression analyses, TyG-BMI failed to exhibit any meaningful linear correlation with 90-day poor outcome in the overall AIS cohort. RCS analyses, however, identified a nonlinear effect of TyG-BMI on 90-day poor outcome. An inflection point in this association was determined at a TyG-BMI value of 193.59. For TyG-BMI values less than or equal to 193.59, a negative effect of TyG-BMI on 90-day poor outcomes (OR = 0.65, 95% CI: 0.48–0.89). In contrast, for TyG-BMI values exceeding this threshold, the effect of TyG-BMI on 90-day unfavorable outcomes was not statistically significant (OR = 1.16, 95% CI: 0.93–1.45, *p* = 0.1765).

**Conclusion:**

Our findings indicate a nonlinear relationship and threshold effect between the TyG-BMI and 90-day unfavorable outcomes in patients with AIS. Only when TyG-BMI ≤ 193.59, the TyG-BMI suggested a negative association with 90-day unfavorable outcomes.

## Introduction

AIS represents a foremost contributor to global mortality and enduring neurological impairment, placing substantial demands on healthcare infrastructure and broader society (1). A significant number of AIS patients continue to encounter unfavorable clinical outcomes, including neurological decline, recurrent cerebrovascular events, and mortality, despite advancements in immediate therapeutic interventions and subsequent prophylactic strategies (2). Therefore, identifying reliable prognostic markers is crucial for effective risk stratification and individualized treatment planning within this patient population.

Recent investigations have increasingly highlighted the effect of IR on poor outcomes after a stroke. The triglyceride-glucose (TyG) index has emerged as a dependable proxy marker for IR (3), exhibiting a robust correlation with the homeostasis model assessment of IR (HOMA-IR) across diverse age groups and ethnicities (3–6). Prior studies have confirmed the TyG index’s ability to predict cardiovascular conditions, such as acute myocardial infarction, coronary artery disease, hypertension, stroke, and heart failure (7–11). Importantly, the combination of the TyG index with obesity indicators, including body mass index (BMI) and waist circumference, has markedly enhanced the precision of IR evaluation (12). In particular, TyG-BMI, the combination of TyG index and BMI, showed good agreement with HOMA-IR in the assessment of IR (12), and is strongly linked to conditions like diabetes, hypertension, prediabetes, nonalcoholic fatty liver disease, and stroke (13–16).

Recognizing the significant effect of IR and stroke outcomes, prior studies have reported inconsistent associations with post-stroke mortality. Some investigations found that higher IR is linked an increased risk of death, whereas others observed a protective relationship (17–19). Therefore, we hypothesize that the TyG-BMI could be a dependable predictor of adverse clinical outcomes in AIS. Despite the significance of this topic, the existing literature contains a paucity of studies examining the connection between TyG-BMI and stroke prognosis. Furthermore, the findings from these limited studies have been inconsistent (20–23). Furthermore, the potential nonlinear connection between TyG-BMI and stroke prognosis has yet to be investigated. To address these gaps, our study aims to explore the effect of TyG-BMI on 90-day poor outcomes in AIS individuals, drawing upon a comprehensive public dataset originating from Korea.

## Methods

### Study design and data source

This study was designed as a retrospective secondary analysis of a prospectively collected cohort dataset. We utilized data derived from a stroke registry system in Korea, with original patient enrollment spanning from January 2010 to December 2016. The investigation focused on assessing 90-day outcomes as the dependent variable, with the TyG-BMI serving as the independent variable in patients with AIS. This study is a secondary analysis of a publicly available dataset published by Kang MK et al. (24). The raw dataset analyzed in this study is publicly available. It was originally collected and published by Kang MK et al. in PLOS ONE. In strict accordance with the Creative Commons Attribution open-access license under which the original data were disseminated, we conducted this secondary analysis without requiring a direct collaboration agreement, ensuring that full appropriate credit and citation are accorded to the original investigators. The original dataset fits our study well. The original study included AIS patients and recorded their 90-day modified Rankin Scale (mRS) scores matching our study needs. The researchers studied the geriatric nutritional risk index and recorded patient height, weight, fasting blood glucose and triglycerides at admission.

### Study population

The primary investigation received approval from the Institutional Review Board at Seoul National University Hospital, with exemption from obtaining individual patient consent (24). For this subsequent data evaluation, we adhered to fundamental ethical guidelines outlined in the Helsinki Declaration while ensuring full compliance with relevant professional codes and regulatory frameworks.

**Figure 1:**
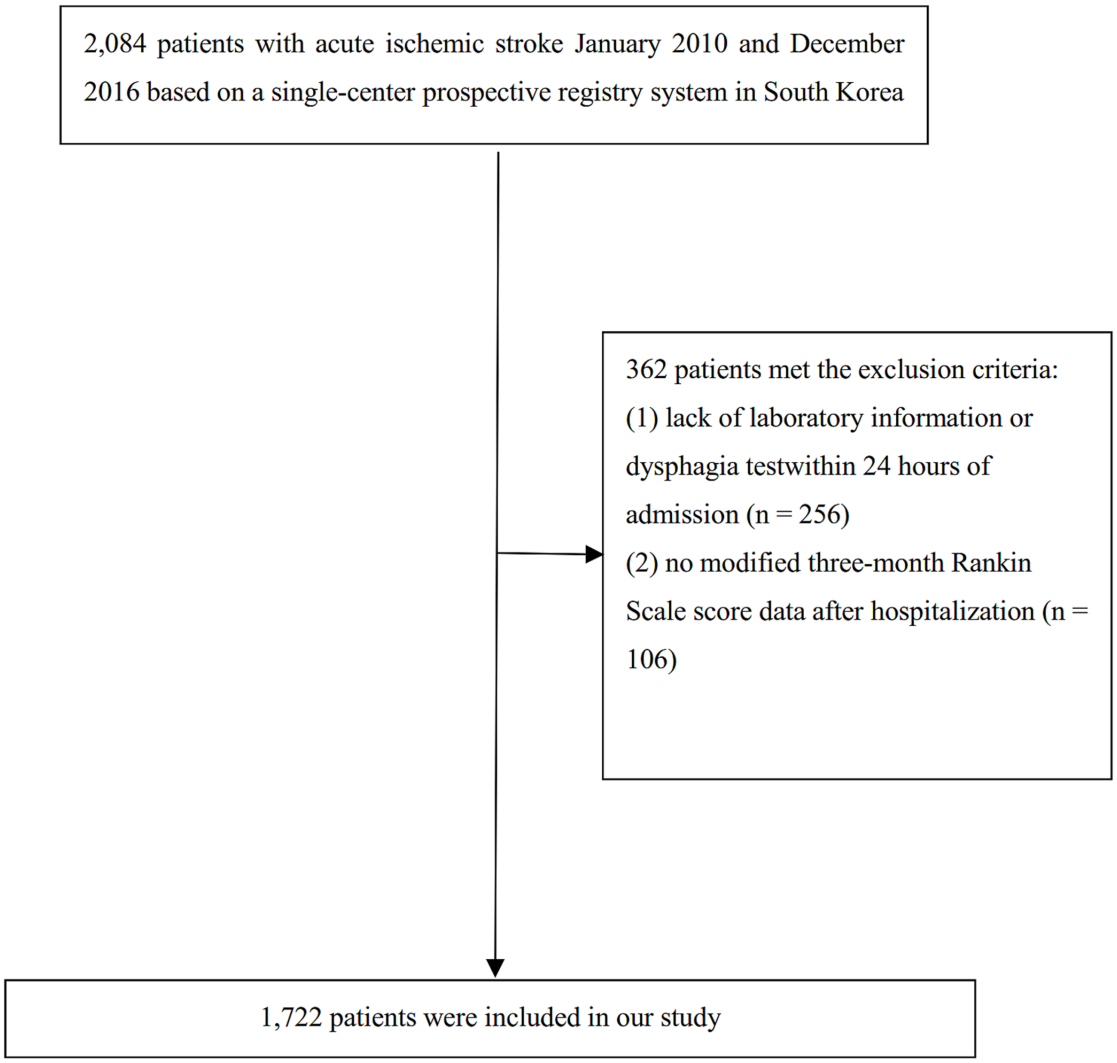
Flowchart of study participants.

AIS was defined and diagnosed based on a clinical diagnosis of AIS with confirmation by brain neuroimaging (CT and/or MRI) at presentation. Individuals were eligible for inclusion if they (1) met the AIS diagnosis criteria, (2) were admitted no more than 7 days after symptom onset. At baseline, 2,084 AIS individuals were included in the study. Exclusion criteria comprised (1) missing laboratory data and/or absence of a dysphagia assessment within 24 h of admission (*n* = 256), and (2) unavailable 90-day mRS scores (*n* = 106). Consequently, the final analytical cohort comprised 1,722 participants (Figure 1).

### Covariates

We identified pertinent covariates informed by prior studies and clinical expertise. These covariates included: (1) Categorical variables: sex, coronary heart disease (CHD), smoking status, diabetes mellitus (DM), stroke etiology, age, and hypertension; (2) Continuous variables: hemoglobin, total cholesterol (TC), white blood cell count (WBC), high-density lipoprotein cholesterol (HDL-C), National Institutes of Health Stroke Scale (NIHSS) score, alkaline phosphatase (ALP), serum creatinine (Scr), serum albumin (ALB), aspartate transaminase (AST), low-density lipoprotein cholesterol (LDL-C), C-reactive protein, alanine transaminase (ALT), and hemoglobin (HGB).

### Data collection

Details concerning the data collection procedures were obtained from the principal investigator. Upon hospital admission, trained nursing personnel employed an automated scale to measure the height and weight of individuals. For stroke patients unable to stand, weight was determined using a scale positioned beneath the bed, and height was measured with a tape measure. Various variables, such as triglycerides (TG), hypertension, stroke etiology, sex, fasting plasma glucose (FPG), CHD, hypertension, and DM, were retrieved from the electronic medical record. All measurements and data extractions were performed according to institutional protocols to ensure consistency and data quality. In addition, DM was defined by glycated hemoglobin ≥6.5%.

### TyG-BMI

TyG-BMI has been widely used and validated as a surrogate marker of IR, and a higher TyG BMI typically leads to greater IR (12). The TyG-BMI was calculated using the following formula: TyG-BMI=BMI (kg/m^2^) × ln [FPG (mg/dL) × TG (mg/dL)/2] (14, 20).

### Outcomes

The evaluation of clinical outcomes was conducted utilizing the mRS score at 90 days following the onset of AIS (24). Data collection was carried out through telephonic interviews or structured outpatient consultations (24). Subjects were stratified into two groups based on their outcomes: good and poor. Poor outcomes were defined as a modified Rankin Scale score ranging from 3 to 6 (24, 25).

### Missing data processing

In this study, which included a cohort of 1,722 patients, missing data were documented and quantified as follows: TC was missing for one patient (0.06%), LDL-C for eight patients (0.46%), and C-reactive protein for 236 patients (13.71%). Missing data for TC, LDL-C, CRP (13.71%) were assumed to be Missing at Random. Standard deviations of these variables in the imputed datasets were highly consistent with those in the original observed dataset, with no significant distributional shifts or statistical differences (Supplementary Table S2). To mitigate the potential negative impact of these missing values and enhance the methodological robustness of our analysis, we employed multiple imputation techniques during the modeling phase (26, 27). The imputation model incorporated variables including HDL-C, NIHSS score, smoking status, AST, C-reactive protein, ALP, HGB, WBC, Scr, DM, ALB, CHD, LDL-C, ALT, age, stroke etiology, hypertension, TC, and sex. A linear regression method, executed over ten iterations, was utilized for the imputation process. The analysis of missing data was conducted within the conceptual framework of Missing-at-Random (27).

### Statistical analysis

Descriptive statistics are reported as mean ± standard deviation (SD) for approximately normally distributed continuous variables, median (interquartile range [IQR]) for skewed continuous variables, and number (percentage) for categorical variables. Group differences were assessed using the ANOVA or Kruskal-Wallis test, or the chi-square test, as appropriate.

Using multivariable logistic regression analysis, we explored the relationship between the TyG-BMI and 90-day poor outcomes in patients with AIS. Model adjustments were guided by clinical expertise and consistent with findings from previous epidemiological studies. Model 1 was unadjusted, while Model 2 accounted for adjustments in smoking status, gender, and age. Model 3 further incorporated adjustments for variables including HDL-C, NIHSS score, AST, WBC, C-reactive protein, ALP, HGB, Scr, DM, CHD, ALB, LDL-C, ALT, stroke etiology, and hypertension. Odds ratios (OR) with 95% confidence intervals (CI) were computed to adjust for potential confounders, as identified in previous studies (20–23, 28–30). Moreover, due to issues of collinearity, TC was excluded from the final model (Supplementary Table S1).

We applied RCS analysis to test potential nonlinear relationships between cardiometabolic indices and 90-day poor outcomes. When evidence of non-linearity was detected, an inflection point was identified using a recursive algorithm, and piecewise logistic regression models were fitted on either side of this threshold.

Our study also performed some sensitivity analyses in order to evaluate the stability of the relationship between TyG-BMI and 90-day poor outcomes. Considering the significant association of DM and CHD with poor outcomes in AIS individuals, we carried out sensitivity analyses by excluding patients with these conditions.

Statistical analyses were conducted using Empower Stats (version 5.2), with a threshold for statistical significance set at *p* < 0.05.

## Results

### Baseline characteristics

Baseline characteristics are summarized in Table 1. The final analysis included a total of 1,722 individuals, with males constituting 61.56% of the cohort. Of these, 106 patients (6.10%) were lost to follow-up. Among these participants, 393 (22.82%) were below the age of 60, 457 (26.54%) were aged between 60 and 70 years, 610 (35.42%) fell within the 70 to 80-year age bracket, and 262 (15.21%) were over 80 years of age. Participants were categorized into subgroups based on the TyG-BMI quartiles. Compared to participants with higher TyG-BMI levels (Q2, Q3, and Q4), those in the low TyG-BMI group (Q1) exhibited lower levels of BMI, TC, TG, LDL-C, FPG, AST, ALT, ALB, Scr, and HGB. Moreover, the Q1 group had a higher proportion of females and lower prevalence rates of smoking, hypertension, and diabetes. Conversely, the Q1 group displayed increased levels of age, HDL-C, ALP, and NIHSS scores.

**Table 1:** The baseline characteristics of participants.

TyG-BMI	Q1 (≤177.68)	Q2 (177.68–198.78)	Q3 (198.78–221.92)	Q4 (>221.92)	*p*-value
Participants	431	430	430	431	
Gender					0.002
Male	241 (55.92%)	251 (58.37%)	288 (66.98%)	280 (64.97%)	
Female	190 (44.08%)	179 (41.63%)	142 (33.02%)	151 (35.03%)	
Age (years)					<0.001
<60	72 (16.71%)	74 (17.21%)	107 (24.88%)	140 (32.48%)	
60 to <70	99 (22.97%)	103 (23.95%)	127 (29.53%)	128 (29.70%)	
70 to <80	157 (36.43%)	177 (41.16%)	143 (33.26%)	133 (30.86%)	
≥80	103 (23.90%)	76 (17.67%)	53 (12.33%)	30 (6.96%)	
BMI (kg/m^2^)	19.73 ± 1.73	22.60 ± 1.26	24.41 ± 1.36	27.29 ± 2.36	<0.001
Smoking status					0.001
No	283 (65.66%)	264 (61.40%)	242 (56.28%)	231 (53.60%)	
Yes	148 (34.34%)	166 (38.60%)	188 (43.72%)	200 (46.40%)	
NIHSS score	4 (2–10)	3 (1–7)	3 (1–6)	3 (1–5)	<0.001
Hypertension					<0.001
No	209 (48.49%)	157 (36.51%)	155 (36.05%)	117 (27.15%)	
Yes	222 (51.51%)	273 (63.49%)	275 (63.95%)	314 (72.85%)	
CHD					0.434
No	389 (90.26%)	382 (88.84%)	374 (86.98%)	377 (87.47%)	
Yes	42 (9.74%)	48 (11.16%)	56 (13.02%)	54 (12.53%)	
DM					<0.001
No	330 (76.57%)	295 (68.60%)	310 (72.09%)	250 (58.00%)	
Yes	101 (23.43%)	135 (31.40%)	120 (27.91%)	181 (42.00%)	
Stroke etiology					0.018
SVO	118 (27.38%)	142 (33.02%)	138 (32.09%)	166 (38.52%)	
LAA	73 (16.94%)	83 (19.30%)	96 (22.33%)	86 (19.95%)	
CE	132 (30.63%)	102 (23.72%)	100 (23.26%)	99 (22.97%)	
Other determined	42 (9.74%)	38 (8.84%)	38 (8.84%)	25 (5.80%)	
Undetermined	66 (15.31%)	65 (15.12%)	58 (13.49%)	55 (12.76%)	
TC (mg/dL)	172.42 ± 38.60	177.95 ± 44.43	181.38 ± 42.72	188.47 ± 45.70	<0.001
TG (mg/dL)	80.26 ± 29.23	95.88 ± 40.61	114.47 ± 45.59	153.65 ± 70.42	<0.001
HDL-C(mg/dL)	50.71 ± 14.48	48.31 ± 14.78	45.10 ± 12.11	42.61 ± 10.93	<0.001
LDL-C (mg/dL)	102.01 ± 32.44	106.85 ± 36.92	111.32 ± 37.76	114.73 ± 40.59	<0.001
FPG (mg//dL)	94.63 ± 30.69	101.33 ± 31.00	107.98 ± 35.70	122.33 ± 46.19	<0.001
AST (U/L)	22 (19–28)	22 (18–29)	23 (18.25–29)	24 (19–31)	0.042
ALT (U/L)	16 (12–21)	17 (13–24)	19 (14–26)	22 (15–33)	<0.001
ALP (U/L)	71 (57–89)	70 (58–85.75)	67 (56–83)	67 (55–80)	0.017
ALB (g/dL)	3.93 ± 0.42	4.01 ± 0.44	4.08 ± 0.41	4.11 ± 0.37	<0.001
Scr (mg/dL)	0.86 (0.70–1.05)	0.87 (0.72–1.07)	0.89 (0.75–1.05)	0.92 (0.77–1.12)	<0.001
HGB (g/dL)	12.73 ± 1.93	13.42 ± 1.96	13.81 ± 1.83	14.26 ± 1.78	<0.001
WBC (10^9^/L)	8.09 ± 3.14	7.99 ± 2.98	7.99 ± 2.69	8.47 ± 2.64	0.047
C-reactive protein (mg/dL)	0.19 (0.05–1.05)	0.15 (0.05–0.69)	0.16 (0.06–0.67)	0.16 (0.06–0.54)	0.619

Values are *n* (%) or mean ± SD or median (quartile). TyG-BMI, triglyceride glucose-body mass index; BMI, body mass index; TC, total cholesterol; TG, triglyceride; LDL-C, low-density lipoproteins cholesterol; HDL-C, high-density lipoprotein cholesterol; FPG: fasting plasma glucose; AST, aspartate aminotransferase; ALT, alanine aminotransferase; ALP, alkaline phosphatase; ALB, serum albumin; Scr, serum creatinine; CHD, coronary heart disease; DM, diabetes mellitus; NIHSS, national institute of health stroke scale; SVO, small vessel occlusion; LAA, large artery atherosclerosis; CE, cardio embolism; HGB, hemoglobin; WBC, white blood cell.

### The prevalence rate of 90-day poor outcomes in individuals with AIS

Table 2 reveals that 469 participants experienced poor outcomes at 90 days, yielding an overall prevalence rate of 27.24%. The prevalence of these outcomes within the TyG-BMI quartile groups was 37.82% in Q1, 26.05% in Q2, 22.56% in Q3, and 22.51% in Q4. It is noteworthy that there was a significantly higher prevalence of 90-day poor outcomes among individuals with lower TyG-BMI levels compared to those with higher TyG-BMI levels.

**Table 2:** Prevalence rate of 90-day poor outcomes in AIS patients.

TyG-BMI	Participants	Participants with poor outcomes	Prevalence rate (95% CI) (%)
Total	1722	469	27.24 (25.13–29.34)
Q1	431	163	37.82 (33.22–42.42)
Q2	430	112	26.05 (21.88–30.21)
Q3	430	97	22.56 (18.59–26.52)
Q4	431	97	22.51 (18.55–26.46)

TyG-BMI, triglyceride glucose-body mass index; AIS, acute ischemic stroke; CI, confidence.

### Results of multivariate analysis

Three separate logistic regression models were conducted to ascertain the independent effect of TyG-BMI on poor outcomes within 90 days (Table 3). In Model 1, a one SD increase in TyG-BMI was demonstrably linked to a 22% reduction in the hazard for adverse events at 90 days (OR = 0.78; 95%CI: 0.70–0.87; *p* < 0.0001). Model 2 revealed a 13% decrease in risk per 1-SD increase in TyG-BMI (OR = 0.87; 95% CI: 0.77–0.97). Conversely, in Model 3, the effect of TyG-BMI and 90-day poor outcomes did not achieve statistical significance (OR = 0.94; 95% CI: 0.80–1.09; *p* = 0.4026).

**Table 3:** Relationship between TyG-BMI and 90-day poor outcomes in AIS.

Exposure	Model 1 (OR, 95%CI, P)	Model 2 (OR, 95%CI, P)	Model 3 (OR, 95%CI, P)
TyG-BMI (per 1-SD increase)	0.78 (0.70, 0.87) < 0.0001	0.87 (0.77, 0.97) 0.0149	0.94 (0.80, 1.09) 0.4026
TyG-BMI group			
Q1	Ref	Ref	Ref
Q2	0.58 (0.43, 0.77) 0.0002	0.60 (0.44, 0.81) 0.0008	0.63 (0.44, 0.91) 0.0135
Q3	0.48 (0.36, 0.65) < 0.0001	0.57 (0.42, 0.77) 0.0003	0.68 (0.47, 0.99) 0.0453
Q4	0.48 (0.35, 0.64) < 0.0001	0.61 (0.45, 0.83) 0.0020	0.76 (0.51, 1.15) 0.1927
P for trend	<0.0001	0.0013	0.2026

Model 1: we did not adjust any covariates. Model 2: we adjusted sex, smoking status, and age. Model 3: we adjusted sex, smoking status, age, HDL-C, LDL-C, ALT, AST, ALP, ALB, Scr, HGB, WBC, C-reactive protein, DM, CHD, hypertension, stroke etiology, and NIHSS score. TyG-BMI, triglyceride glucose-body mass index; AIS, acute ischemic stroke; SD, standard deviation; OR,odds ratios; CI, confidence.

Furthermore, TyG-BMI was subsequently transformed from a continuous measurement into a categorical variable and re-evaluated within the model framework. The results from Model 3 suggested that when compared to the reference group Q1, the OR for the quartile groups Q2, Q3, and Q4 were 0.63 (95% CI: 0.44–0.91), 0.68 (95% CI: 0.47–0.99), and 0.76 (95% CI: 0.51–1.15), respectively.

### The detection of nonlinear relationships

**Figure 2:**
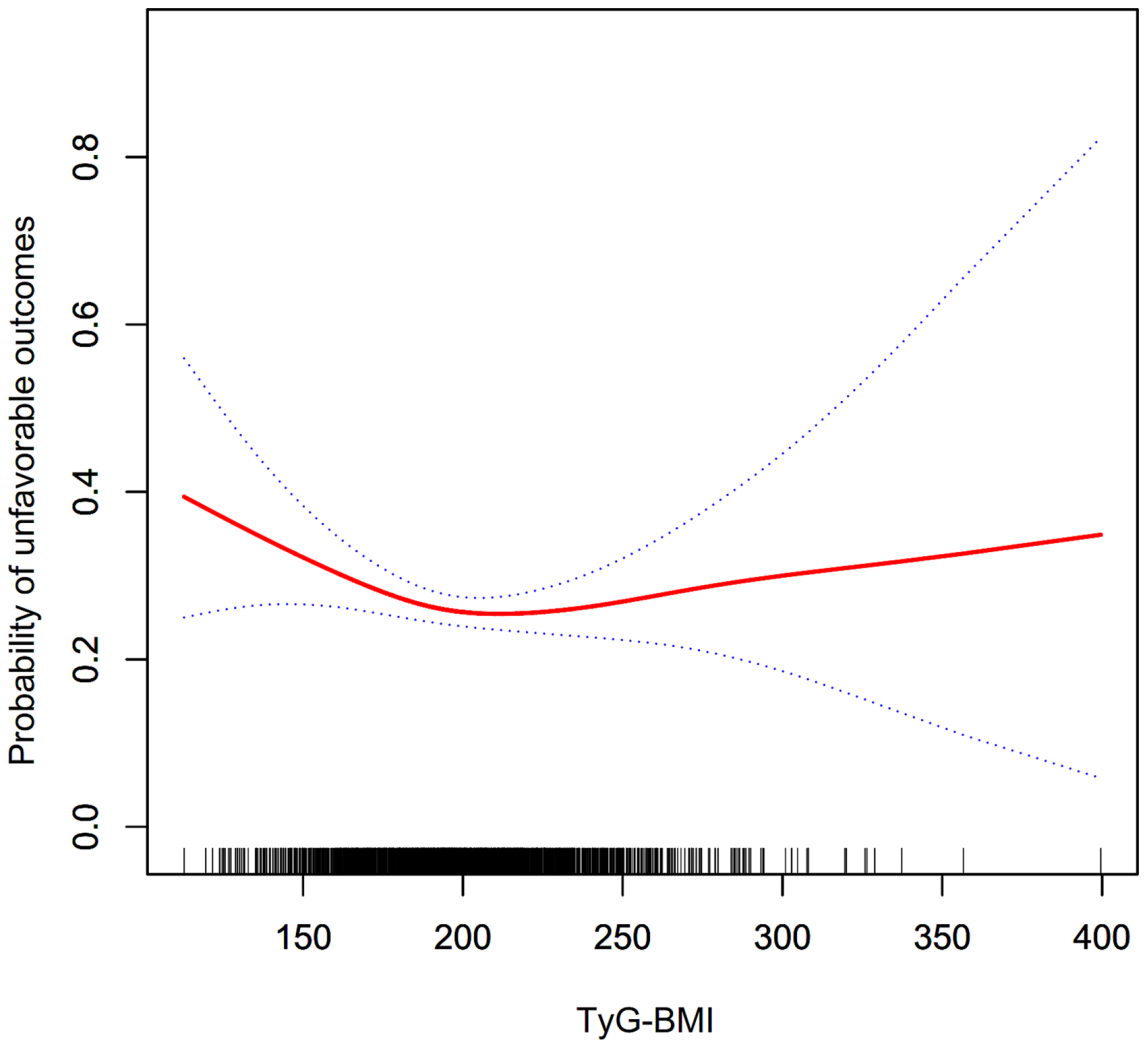
The nonlinear relationship between TyG-BMI and 90-day poor outcomes in AIS patients. A nonlinear relationship was detected in AIS patients after adjusting for sex, smoking status, age, HDL-C, LDL-C, ALT, AST, ALP, ALB, Scr, HGB, WBC, C-reactive protein, DM, hypertension, stroke etiology, and NIHSS score.

Figure 2 and Table 4 depict the nonlinear effect of TyG-BMI on 90-day poor outcome. A recursive method discovered an inflection point at a TyG-BMI value of 193.59. A two-piecewise binary logistic regression model was then employed to compute the OR and corresponding 95%CI for the segments on either side of this threshold. The results demonstrated a significant inverse relationship below the inflection point, with an OR of 0.65 (95% CI: 0.48–0.89). This finding implies that for TyG-BMI values below 193.59, higher TyG-BMI levels are associated with a decreased probability of experiencing poor outcomes at 90 days. In contrast, for TyG-BMI values above the inflection point, the OR was 1.16 (95% CI: 0.93–1.45), suggesting no statistically significant effect of TyG-BMI on poor outcomes.

**Table 4:** The results of a two-piecewise binary logistic regression model.

90-day poor outcomes	OR (95%CI)	*p*
Fitting model by two-piecewise binary logistic regression		
The inflection point of TyG-BMI	193.59	
≤ Inflection point (per 1-SD increase)	0.65 (0.48, 0.89)	0.0067
> Inflection point (per 1-SD increase)	1.16 (0.93, 1.45)	0.1765
P for log-likelihood ratio test	0.009	

We adjusted sex, smoking status, age, HDL-C, LDL-C, ALT, AST, ALP, ALB, Scr, HGB, WBC, C-reactive protein, DM, CHD, hypertension, stroke etiology, and NIHSS score.

### Sensitivity analysis

Table 5 meticulously presents a comprehensive suite of sensitivity analyses to evaluate our findings’ reliability and stability. One sensitivity analysis specifically targeted individuals without DM. After adjusting for potential confounding variables, this analysis confirmed the presence of a nonlinear effect of TyG-BMI on 90-day poor outcomes (Model 4). Similarly, another analysis, which excluded individuals with CHD and accounted for confounding factors, yielded comparable results (Model 5).

**Table 5:** Relationship between TyG-BMI and 90-day poor outcomes in different sensitivity analyses.

90-day adverse clinical outcomes	Model 4 (OR, 95%CI, P)	Model 5 (OR, 95%CI, P)
Fitting model by two-piecewise binary logistic regression		
The inflection point of TyG-BMI	205.1	190.03
≤ Inflection point (per 1-SD increase)	0.72 (0.53, 0.98) 0.0347	0.64 (0.45, 0.91) 0.0138
> Inflection point (per 1-SD increase)	1.33 (0.91, 1.94) 0.1383	1.14 (0.91, 1.43) 0.2559
P for log-likelihood ratio test	0.033	0.018

Model 4 was a sensitivity analysis in participants without DM. We adjusted sex, smoking status, age, HDL-C, LDL-C, ALT, AST, ALP, ALB, Scr, HGB, WBC, C-reactive protein, CHD, hypertension, stroke etiology, and NIHSS score. Model 5 was a sensitivity analysis in participants without CHD. We adjusted sex, smoking status, age, HDL-C, LDL-C, ALT, AST, ALP, ALB, Scr, HGB, WBC, C-reactive protein, DM, hypertension, stroke etiology, and NIHSS score.

## Discussion

In this study, comprising 1,722 adult Korean AIS patients, the nonlinear effect of TyG-BMI on 90-day poor outcomes was identified, with an inflection point at a TyG-BMI value of 193.59. Below this threshold, a statistically significant negative effect of TyG-BMI on 90-day poor outcomes was observed. Conversely, for TyG-BMI values above the inflection point, the relationship with 90-day adverse clinical outcomes lacked statistical significance.

In recent years, accumulating evidence has highlighted the pivotal role of IR in abnormalities of glucose, stroke, and obesity, identifying it as a common pathological feature among these conditions (31). The TyG-BMI integrates FPG, TG, and BMI and has emerged as a robust tool for assessing IR (12). Numerous studies have recently established a strong effect of TyG-BMI on various conditions, including hypertension, diabetes, nonalcoholic fatty liver disease, stroke, and cardiovascular disease (13–15, 32, 33). Nevertheless, the utility of TyG-BMI in evaluating adverse outcomes in AIS individuals remains a subject of debate. A retrospective study involving 456 AIS individuals demonstrated that, after adjusting for variables including age, smoking status, diastolic blood pressure, atrial fibrillation, dyslipidemia, systolic blood pressure, and triglycerides, individuals in the fourth quartile of TyG-BMI, compared to those in the first quartile, exhibited a significantly reduced risk of adverse short-term outcomes (OR: 0.407, 95%CI: 0.185–0.894, *p* = 0.025) (21). Furthermore, a separate study encompassing 1,707 critically ill stroke subjects in intensive care units found that after controlling for confounding factors including gender, age, thrombolysis, thrombectomy, ethnicity, heart failure, diabetes mellitus, hypertension, red blood cell count, white blood cell count, systolic blood pressure, and the sequential organ failure assessment score, elevated TyG-BMI levels were significantly correlated with a decreased risk of 90-day poor outcomes (OR: 0.74, 95% CI: 0.59–0.94, p = 0.02) (20). However, discordant findings exist in the literature. In a study of 1,696 middle-aged and elderly patients with AIS, it was found that an increase in the TyG-BMI significantly heightened the risk of early neurological deterioration (OR: 5.475, 95% CI: 4.157–7.163, *p* < 0.001) (22). Moreover, a retrospective study of 412 patients with AIS after intravenous thrombolysis demonstrated that, after adjusting for confounding variables, there was a significant positive association between TyG-BMI and poor outcomes at three months (OR: 1.080, 95% CI: 1.025–1.138, *p* = 0.004) (23). However, our findings derived from the binary logistic regression model diverge from previous studies’ findings. We hypothesize that this divergence may be due to a nonlinear relationship between TyG-BMI and 90-day poor outcomes. To explore this possibility, we employed GAM and SCF techniques to examine the nonlinear association between TyG-BMI and 90-day poor outcomes. Our current research indicates the presence of a nonlinear relationship and threshold effect involving TyG-BMI and 90-day poor outcomes. Several potential explanations for these discrepancies include: (1) variations in study populations; (2) inadequate investigation of the nonlinear link in previous studies; (3) unlike our study, previous studies may not have accounted for HGB, CHD, ALT, C-reactive protein, stroke etiology, ALP, and ALB, all of which are significantly associated with adverse clinical outcomes, in their analyses of the relationship between TyG-BMI and 90-day adverse outcomes (24, 28–30); and (4) differences in baseline TyG-BMI levels, which may have influenced the outcomes.

Moreover, our investigation revealed a nonlinear effect of TyG-BMI on 90-day poor outcomes. A critical inflection point was identified at a TyG-BMI of 193.59. When the TyG-BMI was less than or equal to this threshold, there was a 35% reduction in the risk of adverse clinical outcomes for each standard deviation increment in TyG-BMI. Conversely, beyond a TyG-BMI of 193.59, this relationship no longer demonstrated statistical significance. Our results suggest that an increase in TyG-BMI correlates with a decreased likelihood of 90-day poor outcomes in patients with AIS. However, this reduction in risk plateaus when the TyG-BMI reaches or exceeds 193.59.

The factors underlying the nonlinear effect of TyG-BMI on 90-day poor outcomes in AIS patients remain elusive. This relationship might be associated with the constituents of the TyG-BMI: FPG, TG, and BMI. Firstly, AIS is characterized by metabolic stress with heightened energy requirements that necessitate the consumption of adipose and muscular tissue (34). TG and adipose tissue are reserves for surplus calories, thus providing essential energy during such metabolic stress. Consequently, low serum levels of TG and BMI could indicate malnutrition, which may hinder neurological recovery (35–37). Furthermore, acute hyperglycemia is often deemed protective, potentially enhancing cellular resistance to ischemic and hypoxic injury (38, 39). However, there is some evidence that acute hyperglycemia is typically associated with worse stroke outcomes, including higher mortality and poorer functional recovery (40–42). In addition, the absence of a statistically significant effect of TyG-BMI on 90-day poor outcomes beyond a threshold of 193.59 might be explained by the presence of energy and nutritional reserves that surpass the fundamental requirements for neural repair in individuals with TyG-BMI values exceeding this limit.

Our study possesses several distinct advantages. Primarily, it delves into the nonlinear relationship between TyG-BMI and the incidence of adverse clinical outcomes in patients with AIS. We applied rigorous statistical techniques to address and mitigate residual confounding effects, thereby enhancing our conclusions’ robustness. Furthermore, we conducted an extensive series of sensitivity analyses to reinforce the reliability of our findings. These analyses involved categorizing TyG-BMI as a categorical variable and re-evaluating the association between TyG-BMI and adverse outcomes after excluding participants with CHD or DM.

However, our study is subject to several limitations. Firstly, the cohort was exclusively composed of Korean individuals and does not contain detailed information on participants’ ethnicity, genetic ancestry, or related subgroup identifiers, which may limit the generalizability of the findings to other populations. Secondly, because this is a secondary analysis of a pre-existing dataset, we lacked data on whether patients received acute reperfusion therapies (specifically intravenous thrombolysis and mechanical thrombectomy). Given that these treatments are major independent determinants of 90-day stroke outcomes, the inability to adjust for them in our multivariable models is a significant limitation, and our results must be interpreted with caution as unmeasured confounding may exist. Thirdly, as with any observational study, despite efforts to adjust for known confounders, there remains the possibility of unmeasured or uncontrolled confounding factors, such as medication use, intravenous thrombolysis, mechanical thrombectomy and family history of stroke. In future research, we plan to collaborate with international scholars to gather more comprehensive data on age, medication use, and family history of stroke. Fourthly, the original study did not provide information on the population with swallowing difficulties. In the future, we will design our own research to include patients with swallowing difficulties and further explore the relationship between TyG-BMI and stroke prognosis. Additionally, we aim to validate our findings across diverse populations with varying genetic backgrounds to enhance the robustness and applicability of our results. Furthermore, the original cohort included patients admitted within a broad 7-day window from symptom onset. Because the exact time from stroke onset to baseline blood collection was not available in the dataset, we could not distinguish between patients in the hyperacute phase versus the acute phase. This is a notable limitation, as the hyperacute phase of stroke is often accompanied by dynamic metabolic shifts, such as stress hyperglycemia and lipid fluctuations secondary to sympathetic and neuroendocrine activation. These stress-induced transient changes could introduce variability into the TyG-BMI measurements, potentially confounding the exact representation of a patient’s baseline metabolic or nutritional status. Future prospective studies with tightly controlled, standardized blood collection times (e.g., strictly within 24 h of onset) are warranted to validate our findings.

## Conclusion

The current study elucidates a nonlinear, threshold effect between TyG-BMI and the incidence of 90-day poor outcomes in Korean patients with AIS. Notably, a negative association between TyG-BMI and these poor outcomes is observed only when TyG-BMI ≤ 193.59. This finding is particularly valuable for clinicians, as it offers a reference point for the management of TyG-BMI in AIS patients.

## Data Availability

The datasets presented in this study can be found in online repositories. The names of the repository/repositories and accession number(s) can be found at: https://journals.plos.org/plosone/article?id=10.1371/journal.pone.0228738.

## Glossary

AIS: acute ischemic stroke
ALB: serum albumin
ALP: alkaline phosphatase
ALT: alanine transaminase
AST: aspartate transaminase
BMI: body mass index
CHD: coronary heart disease
CI: confidence intervals
DM: diabetes mellitus
FPG: fasting plasma glucose
HDL-C: high-density lipoprotein cholesterol
HGB: hemoglobin
HOMA-IR: homeostasis model assessment of insulin resistance
IR: insulin resistance
LDL-C: low-density lipoprotein cholesterol
mRS: modified Rankin Scale
NIHSS: National Institutes of Health Stroke Scale
OR: odds ratios
SCF: smooth curve fitting
Scr: serum creatinine
SD: standard deviation
TC: total cholesterol
TG: triglycerides
TyG-BMI: triglyceride glucose-body mass index
WBC: white blood cell count
