# Supplementary figures and images for: Association between triglyceride glucose-body mass index and outcomes in patients with acute ischemic stroke: a retrospective secondary analysis of a prospective Korean cohort
Source: Front Neurol. 2026 May 29;17:1806171. doi: 10.3389/fneur.2026.1806171 (PMC13260259; doi:10.3389/fneur.2026.1806171)

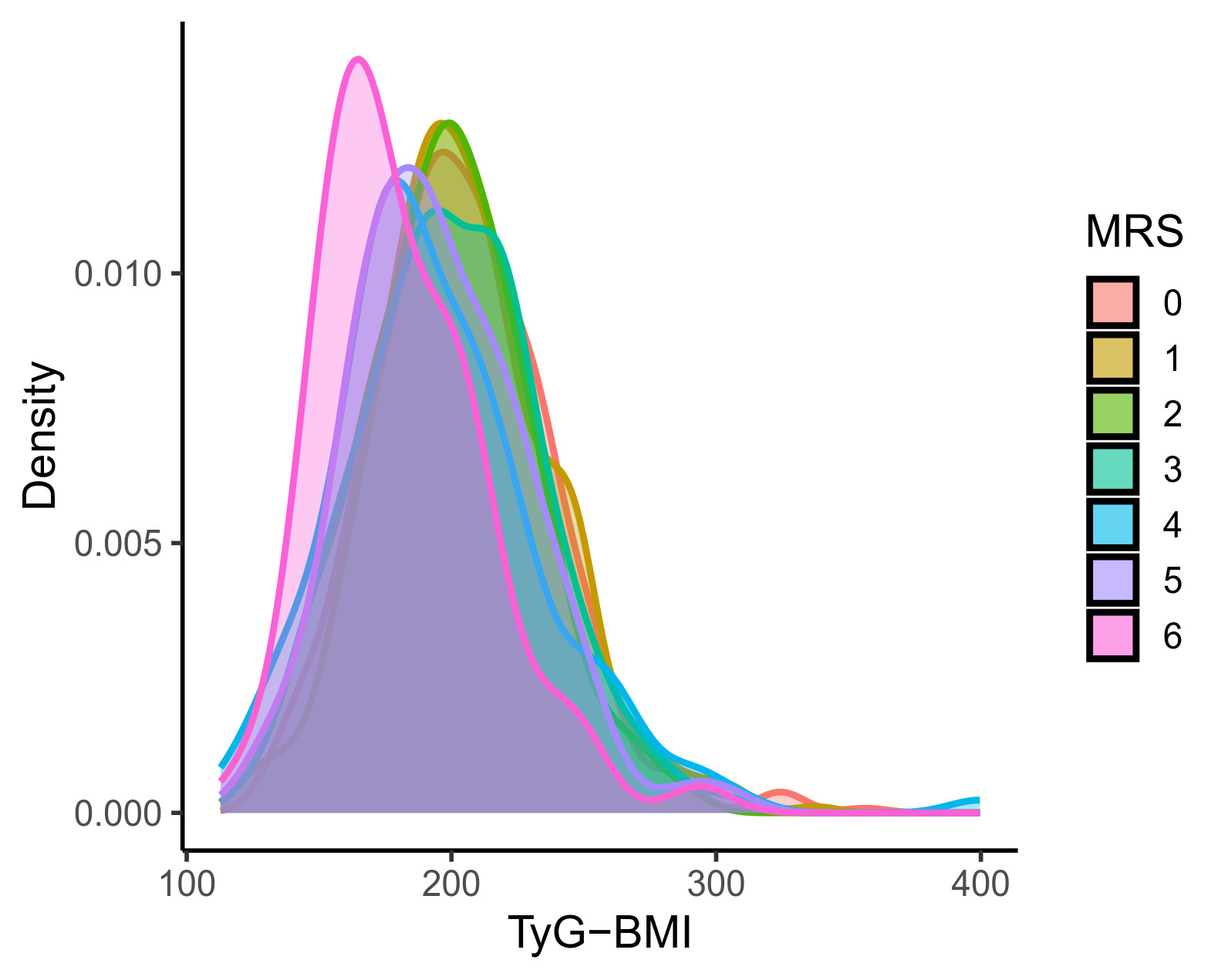

Supplement: Supplementary file 1 [file Image_1.TIF]
