# Supplementary material for: Association between triglyceride glucose-body mass index and outcomes in patients with acute ischemic stroke: a retrospective secondary analysis of a prospective Korean cohort
Source: Front Neurol. 2026 May 29;17:1806171. doi: 10.3389/fneur.2026.1806171 (PMC13260259; doi:10.3389/fneur.2026.1806171)
Supplement: Supplementary file 2 [file Supplementary_file_1.DOCX]

**Table S1** The results of the collinearity screening

|  | Step 1 | Step 2 |
| --- | --- | --- |
| Gender | 1.8 | 1.8 |
| Age | 1.2 | 1.2 |
| Smoking | 1.5 | 1.5 |
| NIHSS score | 1.1 | 1.1 |
| Hypertension | 1.1 | 1.1 |
| CHD | 1.1 | 1.1 |
| DM | 1.1 | 1.1 |
| Stroke etiology | 1.1 | 1.1 |
| ALB | 1.7 | 1.7 |
| TC | 5.8 | NA |
| HDL-C | 1.4 | 1.2 |
| LDL-C | 5.1 | 1.1 |
| AST | 2.0 | 2.0 |
| ALT | 2.0 | 2.0 |
| ALP | 1.1 | 1.1 |
| Scr | 1.2 | 1.1 |
| HGB | 1.8 | 1.8 |
| WBC | 1.2 | 1.2 |
| C-reactive protein | 1.4 | 1.4 |

TC, total cholesterol; LDL-C, low-density lipoproteins cholesterol; HDL-C, high-density lipoprotein cholesterol; AST, aspartate aminotransferase; ALT, alanine aminotransferase; ALP, alkaline phosphatase; ALB, serum albumin; Scr, serum creatinine; CHD, coronary heart disease; DM, diabetes mellitus; NIHSS, national institute of health stroke scale; SVO, small vessel occlusion; LAA, small vessel occlusion; CE, cardio embolism; HGB, hemoglobin; WBC, white blood cell

**Table S2** The baseline characteristics of participants.

| Variables | Before Imputation | After Imputation | P-value |
| --- | --- | --- | --- |
| TC (mg/dL) |  |  |  |
| Mean ± SD | 180.06 ± 43.31 | 180.05 ± 43.30 | >0.05 |
| Median (quartile) | 178.00 (61.00-448.00) | 178.00 (150.00-206.00) | >0.05 |
| LDL-C (mg/dL) |  |  |  |
| Mean ± SD | 108.78 ± 37.30 | 108.73 ± 37.32 | >0.05 |
| Median (quartile) | 105.00 (18.00-355.00) | 105.00 (83.00-131.00) | >0.05 |
| C-reactive protein (mg/dL) |  |  |  |
| Mean ± SD | 1.11 ± 3.08 | 1.05 ± 3.09 | >0.05 |
| Median (quartile) | 0.16 (0.01-30.81) | 0.16 (0.06-0.68) | >0.05 |

TC, total cholesterol; LDL-C, low-density lipoproteins cholesterol

**Table S3** Relationship between TyG-BMI and 90-day poor outcomes in AIS.

| Exposure | Model 1 (OR, 95%CI, P) | Model 2 (OR, 95%CI, P) | Model 3 (OR, 95%CI, P) |
| --- | --- | --- | --- |
| TyG-BMI (per 1-SD increase) | 0.78 (0.70, 0.87) <0.0001 | 0.87 (0.78, 0.98) 0.0189 | 0.94 (0.80, 1.11) 0.4605 |
| TyG-BMI group |  |  |  |
| Q1 | Ref | Ref | Ref |
| Q2 | 0.58 (0.43, 0.77) 0.0002 | 0.59 (0.44, 0.80) 0.0007 | 0.62 (0.42, 0.92) 0.0174 |
| Q3 | 0.48 (0.36, 0.65) <0.0001 | 0.56 (0.41, 0.77) 0.0003 | 0.72 (0.48, 1.08) 0.1085 |
| Q4 | 0.48 (0.35, 0.64) <0.0001 | 0.62 (0.45, 0.84) 0.0023 | 0.77 (0.50, 1.19) 0.2395 |
| P for trend | <0.0001 | 0.0189 | 0.4605 |

Model 1: we did not adjust any covariates.

Model 2: we adjusted sex, smoking status, and age.

Model 3: we adjusted sex, smoking status, age, HDL-C, LDL-C, ALT, AST, ALP, ALB, Scr, HGB, WBC, C-reactive protein, DM, CHD, hypertension, stroke etiology, and NIHSS score.

TyG-BMI, triglyceride glucose-body mass index; AIS, acute ischemic stroke; SD, standard deviation; OR ,odds ratios; CI, confidence

**Table S4** The results of a two-piecewise binary logistic regression model.

| 90-day poor outcomes | OR (95%CI) | P |
| --- | --- | --- |
| Fitting model by two-piecewise binary logistic regression |  |  |
| The inflection point of TyG-BMI | 196.58 | |
| ≤ Inflection point (per 1-SD increase) | 0.66 (0.48, 0.90) | 0.0084 |
| > Inflection point (per 1-SD increase) | 1.22 (0.95, 1.56) | 0.1210 |
| P for log-likelihood ratio test | 0.009 | |

Note: We adjusted sex, smoking status, age, HDL-C, LDL-C, ALT, AST, ALP, ALB, Scr, HGB, WBC, C-reactive protein, DM, CHD, hypertension, stroke etiology, and NIHSS score.

**Table S5** Relationship between TyG-BMI and 90-day poor outcomes in different sensitivity analyses.

| 90-day adverse clinical outcomes | Model 4 (OR ,95%CI, P) | Model 5 (OR ,95%CI, P) | Model 6 (OR ,95%CI, P) |  |
| --- | --- | --- | --- | --- |
| Fitting model by two-piecewise binary logistic regression |  |  |  |  |
| The inflection point of TyG-BMI | 203.77 | 186.49 | 198.91 |  |
| ≤ Inflection point (per 1-SD increase) | 0.60 (0.36, 1.00) 0.0515 | 0.57 (0.37, 0.87) 0.0091 | 0.70 (0.51, 0.94) 0.0194 |  |
| > Inflection point (per 1-SD increase) | 1.19 (0.86, 1.64) 0.2874 | 1.18 (0.95, 1.48) 0.1353 | 1.15 (0.89, 1.48) 0.2899 |  |
| P for log-likelihood ratio test | 0.034 | 0.008 | 0.032 |  |

Model 4 was a sensitivity analysis in female. We adjusted smoking status, age, HDL-C, LDL-C, ALT, AST, ALP, ALB, Scr, HGB, WBC, C-reactive protein, DM, CHD, hypertension, stroke etiology, and NIHSS score.

Model 5 was a sensitivity analysis in participants without age ≥ 80 years. We adjusted sex, smoking status, age, HDL-C, LDL-C, ALT, AST, ALP, ALB, Scr, HGB, WBC, C-reactive protein, DM, CHD, hypertension, stroke etiology, and NIHSS score.

Model 6 was a sensitivity analysis in participants without NIHSS score ≥ 16. We adjusted sex, smoking status, age, HDL-C, LDL-C, ALT, AST, ALP, ALB, Scr, HGB, WBC, C-reactive protein, DM, CHD, hypertension, stroke etiology, and NIHSS score.

**Figure S1** Distribution of triglyceride glucose-body mass index (TyG-BMI) by 3-month Modified Rankin Scale (mRS) Score
